# Aldehyde dehydrogenase serves as a biomarker for worse survival profiles in ovarian cancer patients: an updated meta-analysis

**DOI:** 10.1186/s12905-018-0686-x

**Published:** 2018-12-06

**Authors:** Yan Xia, Xuemin Wei, Hui Gong, Yunxiang Ni

**Affiliations:** Department of Obstetrics and Gynecology, Tongren Hospital, Shanghai Jiao Tong University School of Medicine, 1111 Xianxia Road, Shanghai, 200336 China

**Keywords:** Aldehyde dehydrogenase (ALDH), Overall survival, Disease free survival, Ovarian cancer, Meta-analysis

## Abstract

**Background:**

The purpose of this comprehensive meta-analysis was to assess the association of aldehyde dehydrogenase (ALDH) expression with overall survival (OS) and disease-free survival (DFS)/progression-free survival (PFS) in ovarian cancer patients.

**Methods:**

Systematic searches of Pubmed databases was performed to identify relevant literature published before February 28, 2018. A total of 14 studies (13 articles) with 2210 ovarian cancer patients were pooled. All included studies were performed by using Immunohistochemistry (IHC) for detection of ALDH expression. Hazard ratio (HR) and 95% confidence interval (CI) were extracted from included studies to evaluate the correlation of ALDH expression with OS and DFS/PFS.

**Results:**

High expression of ALDH was associated with worse OS (HR: 1.43; 95% CI: 1.18–1.73) and poor DFS/PFS (HR: 1.55, 95% CI: 1.12–2.14). No evidence of publication bias was observed in OS (Begg’s test, *P* = 0.113; Egger’s test, *P* = 0.355) and DFS/PFS (Begg’s test, *P* = 0.655; Egger’s test, *P* = 0.189) in ovarian cancer patients. The subgroup of studies with cut-off value of low expression showed that high expression of ALDH was correlated with poor OS (HR: 1.36; 95% CI: 1.14–1.62) and DFS/PFS (HR: 1.79; 95% CI: 1.45–2.20) in ovarian cancer patients, with no observed heterogeneity (OS: I^2^ = 0%, *P* = 0.45; DFS/PFS: I^2^ = 0%, *P* = 0.55).

**Conclusion:**

In conclusion, high expression of ALDH is correlated with worse survival profiles in ovarian cancer patients, indicating that ALDH might act as a potential molecular biomarker for prognosis of ovarian cancer.

## Background

Ovarian cancer is one of the common gynecological malignancies featured by several symptoms (including bloating, pelvic pain, abdominal swelling as well as loss of appetite) [1–4]. It has been reported to be the seventh frequently diagnosed cancer and the fourth commonest causes of cancer-related death [1–4]. According to statistical reports, ovarian cancer occurs approximately 239,000 new cases as well as causes estimated 151,900 deaths worldwide in 2012, and happens nearly 52,100 new cases as well as results in 22,500 deaths in China during 2015 [1–4]. Owning to obscure symptoms at an early stage, fast progression, wide metastasis and high recurrence, the prognosis of ovarian cancer patients is far less satisfaction although improvement has been achieved in the disease management (such as imaging, histopathology, surgery as well as chemotherapy) [1]. Hence, further exploration related to additional and convincing biomarkers is imperative to supervise tumor progression for the promotion of prognosis in ovarian cancer patients.

Cancer stem cells (CSCs) are considered as a small class of tumor cells mass with self-renewing capacity, heterogeneity as well as resistance to chemotherapy/ radiotherapy, which play critical roles in tumor initiation, progression and maintenance [5, 6]. As one of the most common markers of CSCs, aldehyde dehydrogenase (ALDH) involves in the tumor development and progression processes of various carcinomas, including breast cancer, colorectal cancer, prostate cancer as well as bladder cancer [7–10]. Also, there are several clinical trials investigating the correlation of ALDH expression with the prognosis in ovarian cancer patients, whereas controversial results still existed [11–13]. Regarding that these controversial results might be caused by relatively small sample size, different inclusion criteria, outcomes assessed measures or other factors in different clinical studies, the comprehensive meta-analyses are needed. Although there is one previous meta-analysis including 1258 ovarian cancer patients from 7 studies (6 articles) in 2013, the number of included patients and studies is still relatively small [14]. Thus we performed additional comprehensive meta-analysis including 14 studies (13 articles) with 2210 ovarian cancer patients, and the purpose was to assess the association of ALDH expression with overall survival (OS) and disease-free survival (DFS)/ progression-free survival (PFS) in ovarian cancer patients.

## Methods

### Literature search strategy

Based on the standard meta-analysis guidelines, we performed systematic searches of the National Center for Biotechnology Information (NCBI) Pubmed databases to identify relevant literature published before February 28, 2018 [15]. The studies were searched by using the following keywords in various combinations: Ovarian AND (neoplasm OR carcinoma OR cancer OR Tumor) AND (ALDH OR Aldehyde Dehydrogenase). Meanwhile, the reference lists of relevant articles were also manually retrieved.

### Selection criteria

The criteria for inclusion were as follows: (1) All patients were diagnosed as ovarian cancer by pathological findings. (2) The association of ALDH expression with OS, DFS or PFS was investigated. (3) ALDH expression was detected by Immunohistochemistry (IHC). The criteria for exclusion were as follows: (1) The studies about animals or cell lines. (2) Articles were reviews, letters, case reports, editorials as well as expert opinions. (3) The studies were lack of essential information related to survival. All articles from the NCBI Pubmed databases are English, and there was no geographic location restriction. This meta-analysis was designed according to the Preferred Reporting Items for Systematic reviews and Meta-Analyses (PRISMA) Statement [16]. This was a meta-analysis, which was not registered on Clinicaltrials.gov or similar repositories.

### Data extraction and quality assessment

The studies were selected, and the data were extracted independently by two authors. The following information was recorded: author’s name, publish year, country, patients size, histology and disease stage, survival data, cut-off value (There were 3 studies set cut-off class as high expression (> 20%), 6 studies defined cut-off class as low expression (≤20%) and 5 studies installed cut-off class as IRS), Hazard ratio (HR), 95% confidence interval (CI), and so on. Survival data included OS, DFS as well as PFS, and there were 13 studies for OS, 6 studies for DFS and 4 studies for PFS. Considering the number of studies related to DFS or PFS was relatively small, which might poor statistical power in this meta-analysis, thus, the survival data was classified as OS and DFS/PFS for final analysis, this classified method was similar with the method from previous study conducted by Feng et al. [17]. Any disagreement was settled by a third reviewer. In addition, the items were treated as “Not available (NA)” if above data were not collected in the original articles. The Newcastle-Ottawa quality assessment scale was utilized to evaluate the methodological quality of studies included in this meta-analysis [18]. This scale refers to the evaluation of patient size, selection, study comparability, follow-ups as well as outcomes. Interpretation of the scale was carried out by stars that subsequently were added up and utilized for the comparison of study quality in a quantitative manner.

### Statistics

All statistical analyses were performed with “meta” package in R. The major outcomes for this meta-analysis were the correlation of ALDH expression with OS and DFS/PFS in ovarian cancer patients. Some studies directly provided HR and 95% CI, while as to some other studies which did not give these data clearly, we used Engauge Digitizer version 4.1 and the relevant methods for calculation from available data or Kaplan-Meier survive cure [19] (free software down-loaded from http://sourceforge.net). In addition, we selected the multivariate analysis if there were univariate analysis and multivariate analysis. Chi-squared test was used for the evaluation of heterogeneity that was assessed by I^2^ value, and I^2^ value < 50% was considered as no or moderate heterogeneity, while I^2^ value > 50% was considered as statistically significant heterogeneity. The significant heterogeneity was detected by using a random-effects model. Otherwise, the fixed effects model was used [20]. The possibility of published bias was assessed by Begg’s test and Egger’s test [21, 22]. The stability of pooled results was examined by Sensitivity analysis. Moreover, HR > 1 was considered as a worse survival, and when the 95% CI did not overlap with 1, the influence of high ALDH expression on survival implied statistically significant. All the *p*-values were two-sided, and *p* < 0.05 was considered statistically significant.

## Results

### Literature search analysis

**Fig. 1:**
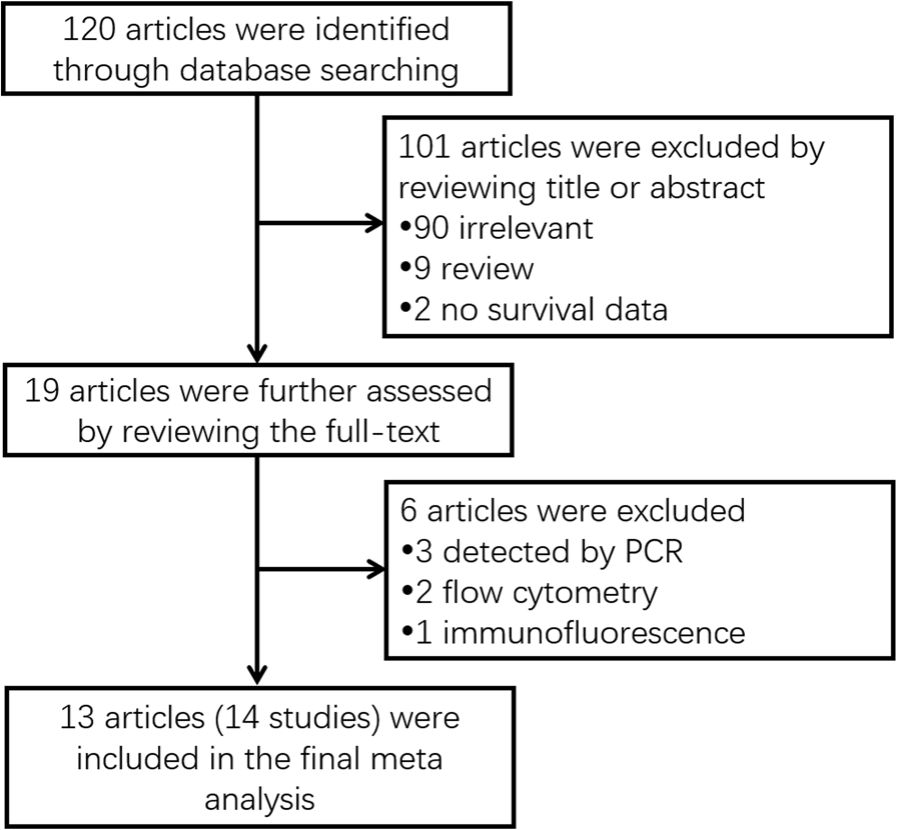
Flow diagram of the studies identification and selection

A total of 120 articles were initially identified as appropriate through database searching, while 101 articles were excluded by reviewing title or abstract, including 90 articles which were irrelevant, 9 articles which were reviews and 2 articles which did not record survival data. The remaining 19 articles were further assessed by reviewing the full-text, whereas 6 articles were excluded, including 3 articles that ALDH was detected by PCR, 2 articles that ALDH was detected by flow cytometry and 1 article that ALDH was detected by immunofluorescence. Finally, there were 13 articles (14 studies) included in the final meta-analysis (Fig. 1).

### Characteristics of the included studies

The main characteristics of 14 studies (13 articles) included in this meta-analysis were summarized in Table 1. All studies were published from 2009 to 2018 with the sample size ranging from 37 to 440. There were 4 studies from China, 4 from USA, 3 from Japan, 2 from Germany and 1 from Norway. In addition, 5 studies defined the cut off value by complex score combining intensity, while others used the percentage of ALDH expression to define the cut off value. Detailed information of these studies was shown in Table 1.

**Table 1:** Basic characteristics of all studies included in this meta-analysis

Author	Country	Year	Sample size	Cutoff	Cutoff class	Survival	HR (95% CI)	HR	HR_low	HR_high	Analysis
Yu	China	2017	207	IRS ≥ 3	IRS	OS	1.86 (1.16–2.98)	1.86	1.16	2.98	Multivariate
Huang	Norway	2015	248	IRS ≥ 7	IRS	OS	0.913 (0.735–1.134)	0.913	0.735	1.134	Multivariate
						PFS	1.023 (0.756–1.165)	1.023	0.756	1.165	Multivariate
Mizuno	Japan	2015	81	≥10%	low	OS	1.765 (0.874–2.984)	1.765	0.874	2.984	Univariate
						PFS	2.134 (1.103–3.654)	2.134	1.103	3.654	Univariate
Madhuchhanda	USA	2017	124	≥5%	low	OS	1.127 (0.903–1.564)	1.127	0.903	1.564	Univariate
						PFS	1.097 (0.874–1.645)	1.097	0.874	1.645	Univariate
Ruscito	Germany	2017	112	IRS ≥ 2	IRS	OS	1.707 (1.012–2.881)	1.707	1.012	2.881	Multivariate
Chen	China	2015	80	≥10%	low	OS	2.684 (1.149–6.249)	2.684	1.149	6.249	Multivariate
Sun	China	2015	100	IRS ≥ 9	IRS	PFS	2.574 (1.297–5.638)	2.574	1.297	5.638	Univariate
Chang	USA	2009	440	> 20%	high	OS	0.92 (0.67–1.27)	0.92	0.67	1.27	Multivariate
						DFS	0.79 (0.61–1.02)	0.79	0.61	1.02	
Deng	USA	2010	439	≥10%	low	OS	1.27 (1.03–1.56)	1.27	1.03	1.56	NA
						DFS	1.77 (1.37–2.29)	1.77	1.37	2.29	
Landen	USA	2010	65	> 1%	low	OS	1.39 (0.69–2.80)	1.39	0.69	2.8	Univariate
						DFS	2.03 (1.16–3.57)	2.03	1.16	3.57	
Wang	China	2012	84	> 50%	high	OS	2.43 (1.12–5.28)	2.43	1.12	5.28	Multivariate
						DFS	1.70 (0.77–3.77)	1.7	0.77	3.77	
Liebscher	Germany	2013	131	IRS ≥ 4	IRS	OS	2.01 (1.03–3.93)	2.01	1.03	3.93	Multivariate
Kuroda	Japan	2013a	62	> 20%	high	OS	1.63 (0.34–7.89)	1.63	0.34	7.89	Univariate
						DFS	1.95 (0.95–4.00)	1.95	0.95	4	
Kuroda	Japan	2013b	37	> 15%	low	OS	3.62 (0.47–27.88)	3.62	0.47	27.88	Univariate
						DFS	3.97 (0.60–26.19)	3.97	0.6	26.19	

*HR* Hazard ratio, *CI* confidence interval, *OS* overall survival, *PFS* progression-free survival, *DFS* disease free survival, *IRS* Infrequent restriction site, *NA* Not available

### Correlation of ALDH expression with OS in ovarian cancer

**Fig. 2:**
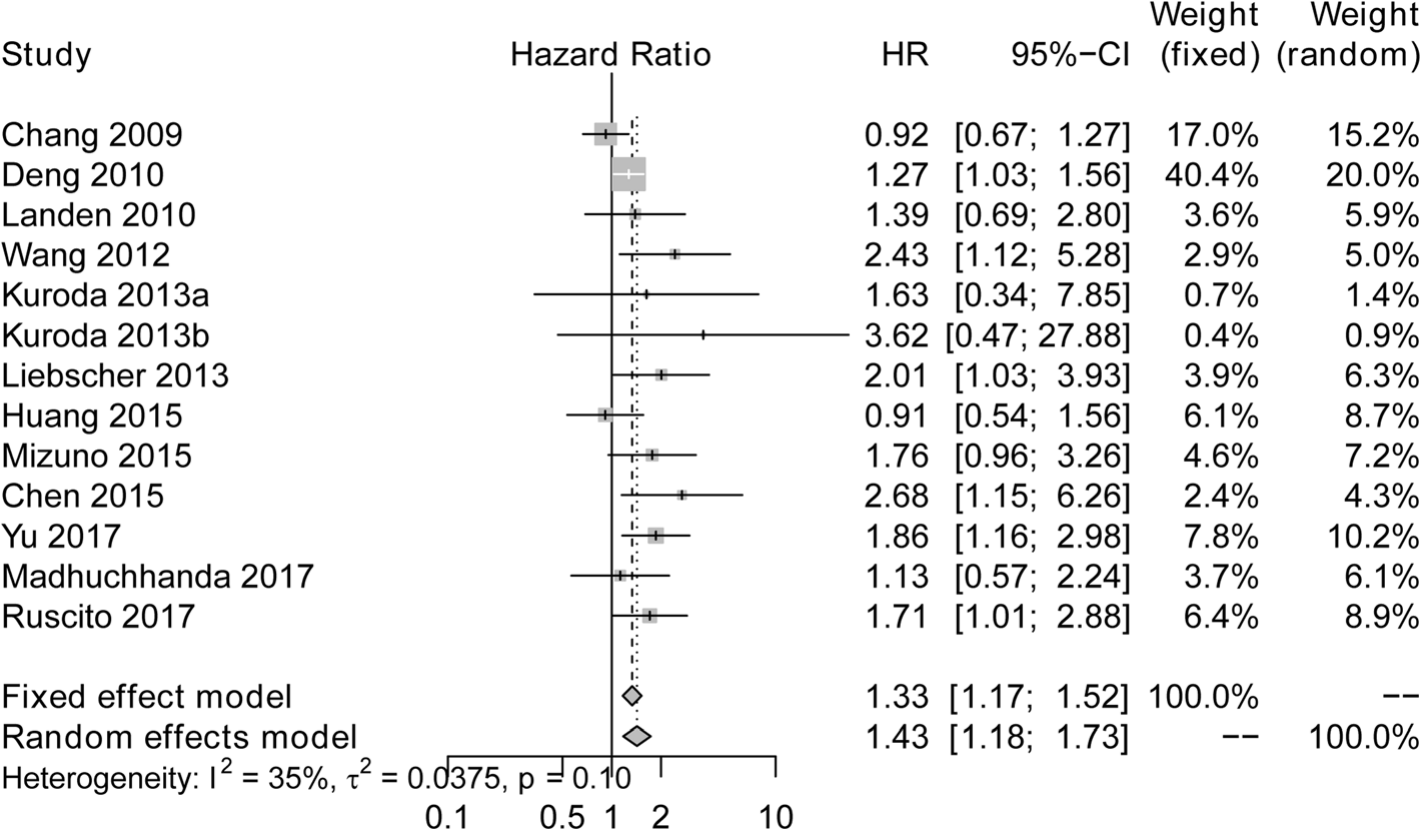
Forest plots of studies assessing the correlation between ALDH expression and OS. ALDH, Aldehyde dehydrogenase; HR, Hazard ratio; CI, confidence interval; OS, overall survival

High expression of ALDH was associated with worse OS (HR:1.43; 95% CI: 1.18–1.73) (Fig. 2). No significant heterogeneity was found among the studies using the fixed effects model (I^2^ = 35%, *P* = 0.10).

### Correlation between ALDH expression with OS in subgroup analysis with different cut-off class

**Fig. 3:**
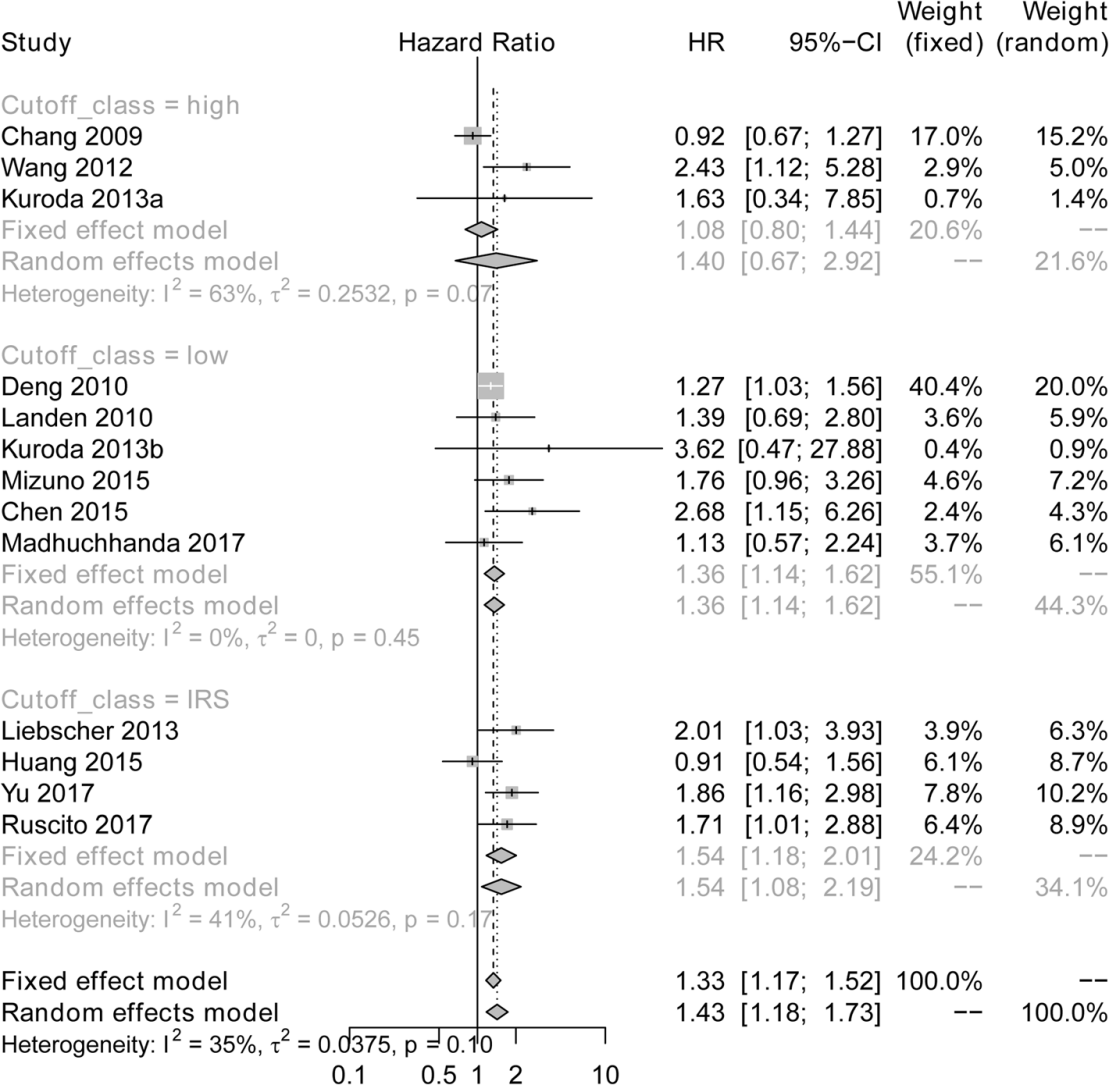
Subgroup meta-analysis of studies with different cut off value assessing the association of ALDH expression and OS. ALDH, Aldehyde dehydrogenase; HR, Hazard ratio; CI, confidence interval; OS, overall survival

The results of subgroup meta-analysis of studies were shown in Fig. 3. The subgroup of studies with cut-off value of low expression (HR: 1.36; 95% CI: 1.14–1.62) and infrequent restriction site (IRS) expression (HR: 1.33; 95% CI: 1.17–1.52) showed that high expression of ALDH was correlated with poor OS in ovarian cancer patients, with no observed heterogeneity (low expression of ANRIL: I^2^ = 0%, *P* = 0.45; IRS expression of ANRIL: I^2^ = 41%, *P* = 0.17). However, the subgroup of studies with cut off value of high expression revealed that there was no association of ALDH expression with OS in ovarian cancer patients (HR: 1.40; 95%CI: 0.67–2.92), and significant heterogeneity existed using the random effects model (I^2^ = 63%, *P* = 0.07).

### Funnel plot to assess publication bias for the association of ALDH and OS in ovarian cancer

**Fig. 4:**
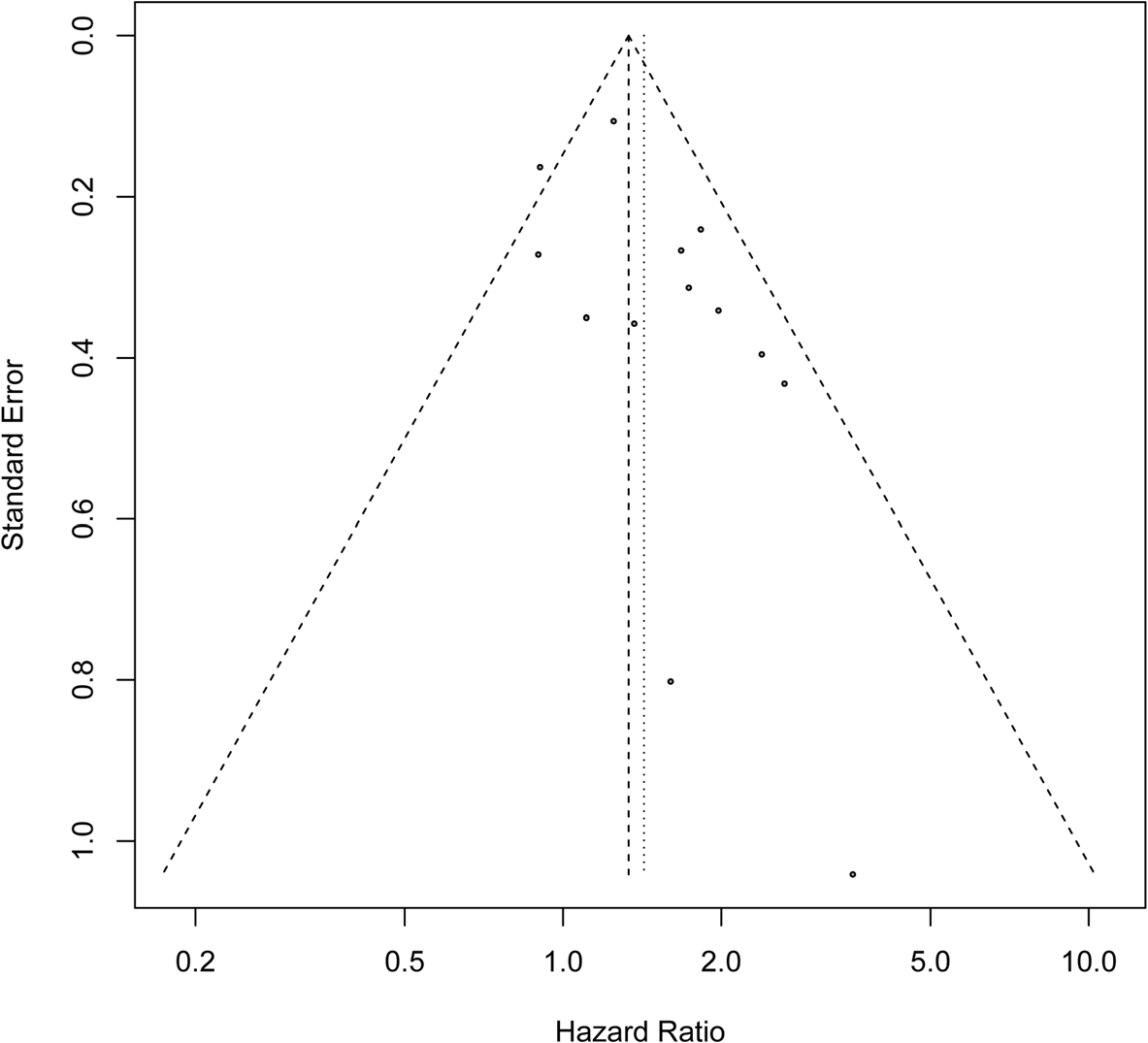
Funnel plot for the publication bias test of included studies for ALDH expression and OS. ALDH, Aldehyde dehydrogenase; OS, overall survival

Publication bias was evaluated by the Begg’s funnel plot and Egger’s test, which showed that the shape of the funnel plot was not obvious asymmetric, indicating no evidence of publication bias in OS of patients with ovarian cancer (Begg’s test, *P* = 0.113; Egger’s test, *P* = 0.355) (Fig. 4).

### Sensitivity analysis of studies evaluating OS

**Fig. 5:**
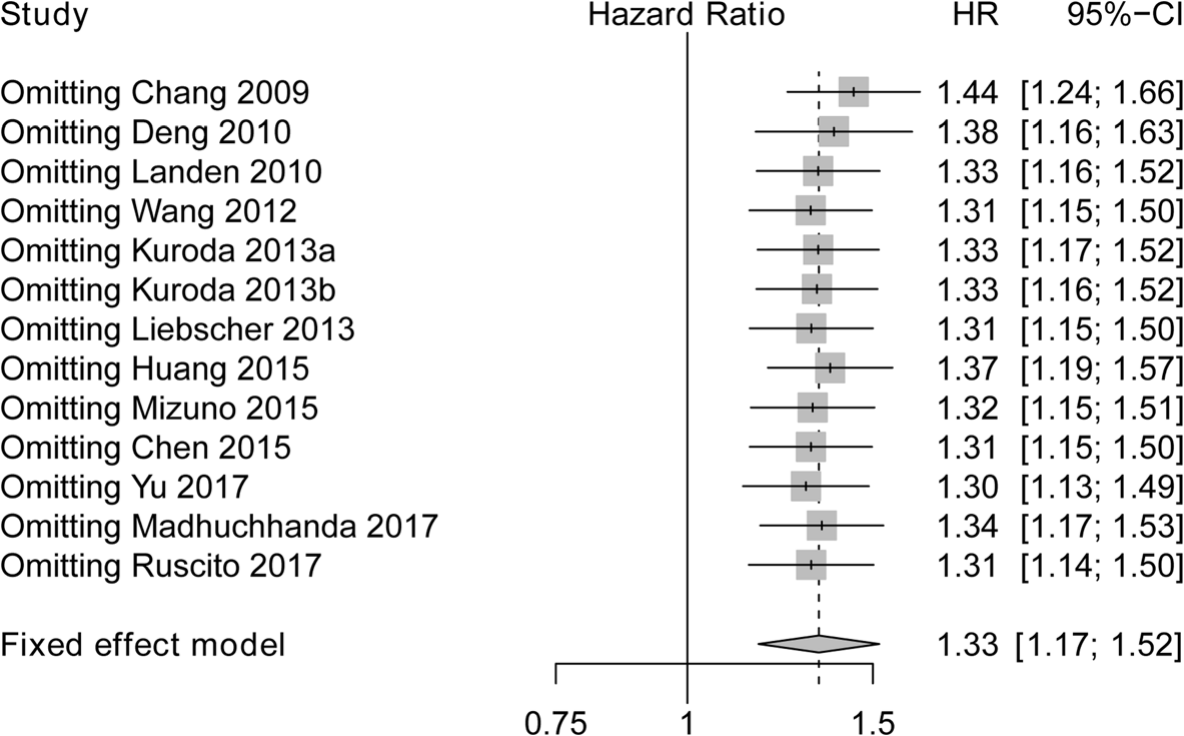
Sensitivity analysis for evaluating the correlation between ALDH expression and OS. ALDH, Aldehyde dehydrogenase; HR, Hazard ratio; CI, confidence interval; OS, overall survival

Sensitivity analysis was performed for the stability of the crude results, which showed that poor HR was not changed, and omitting Chang 2009 had a numerically great impact on OS compared to omitting other studies. Therefore, these results suggested that the conclusion was stable (Fig. 5).

### Correlation of ALDH expression with DFS/PFS in ovarian cancer

**Fig. 6:**
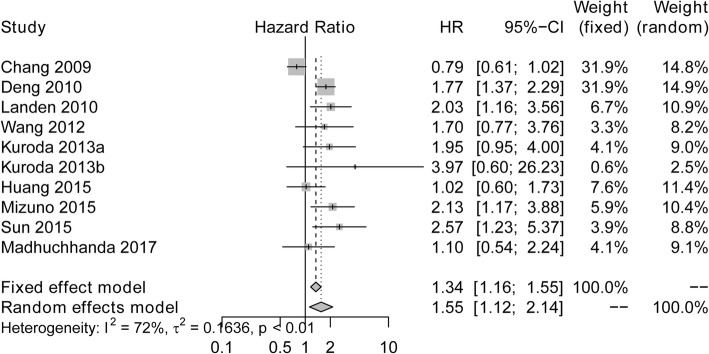
Forest plots of studies assessing the correlation between ALDH expression and DFS/PFS. ALDH, Aldehyde dehydrogenase; HR, Hazard ratio; CI, confidence interval; DFS, disease-free survival; PFS, progression-free survival

As presented in Fig. 6, ALDH upregulation was correlated with poor DFS/PFS (HR: 1.55, 95% CI: 1.12–2.14). Owning to significant heterogeneity among the studies (I^2^ = 72%, *P* < 0.01), the random effects model was performed for the calculation of the pooled HR with corresponding 95% CI.

### Correlation between ALDH expression with DFS/PFS in subgroup analysis with different cut-off class

**Fig. 7:**
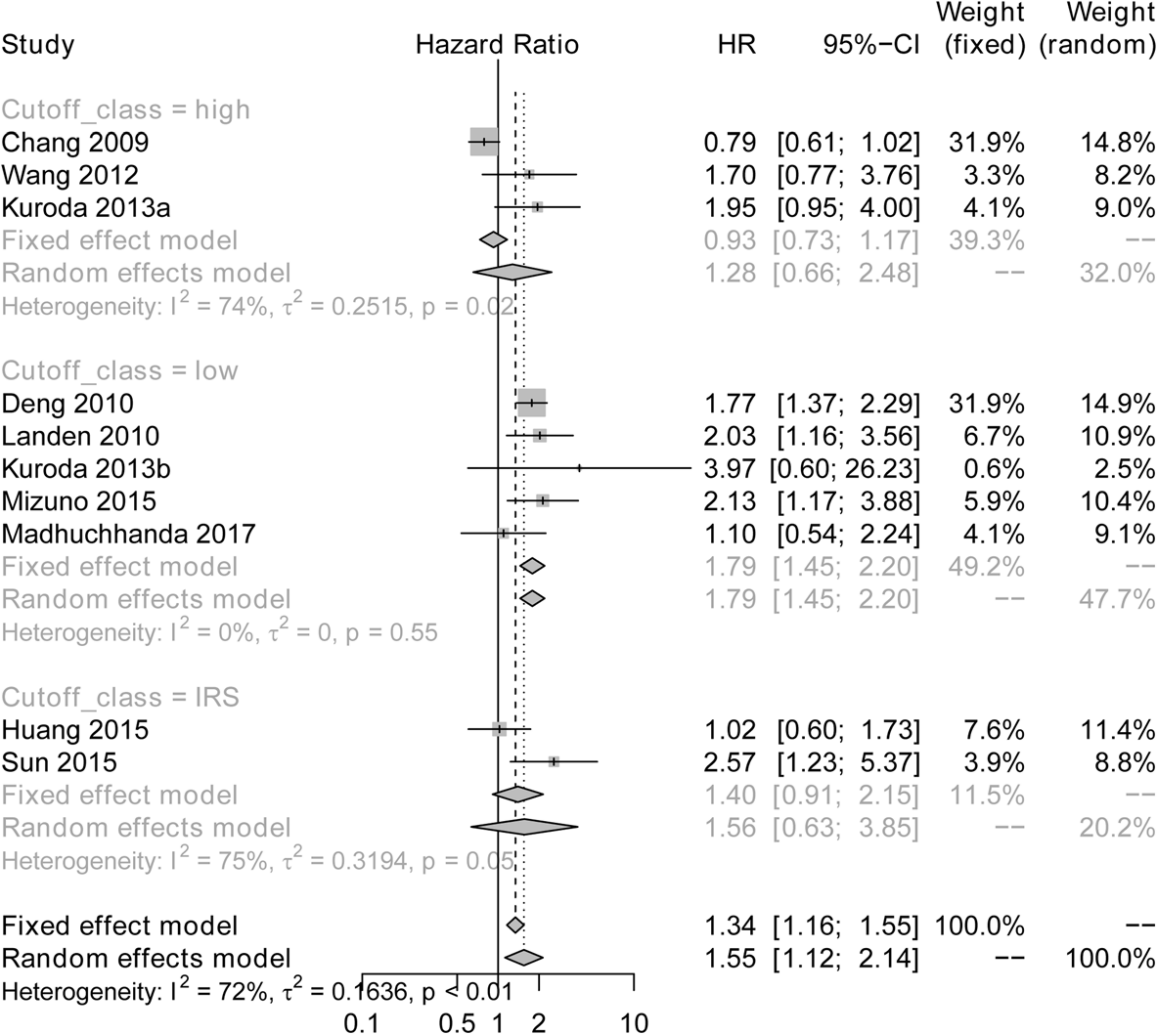
Subgroup meta-analysis of studies with different cut off value assessing the association of ALDH expression and DFS/PFS. ALDH, Aldehyde dehydrogenase; HR, Hazard ratio; CI, confidence interval; DFS, disease-free survival; PFS, progression-free survival

As shown in Fig. 7, subgroup meta-analysis of studies with cut off value of low expression disclosed that high ALDH expression was associated with worse DFS/PFS (HR: 1.79; 95% CI: 1.45–2.20), which was without obvious heterogeneity by using the fixed effects model (I^2^ = 0%, *P* = 0.55). Whereas the subgroups meta-analysis of studies with cut off value of high expression (HR: 1.28; 95% CI: 0.66–2.48) as well as IRS expression (HR: 1.56; 95% CI: 0.63–3.85) illustrated no correlation of ALDH expression with DFS/PFS in ovarian cancer patients, and heterogeneity was observed using the random effects model (high expression of ANRIL: I^2^ = 74%, *P* = 0.02; IRS expression of ANRIL: I^2^ = 75%, *P* = 0.05).

### Funnel plot to assess publication bias for the association of ALDH and DFS/PFS in ovarian cancer

**Fig. 8:**
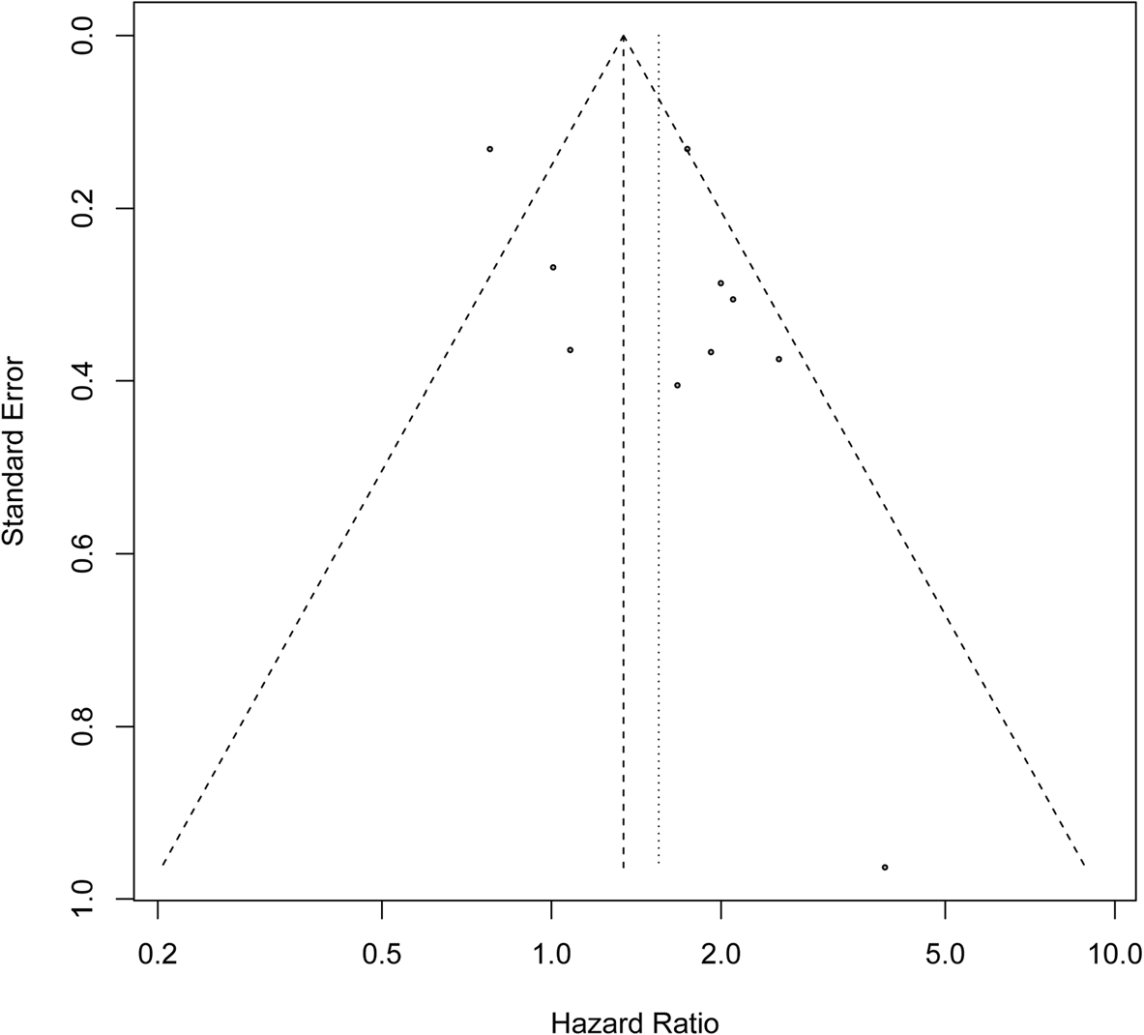
Funnel plot for the publication bias test of included studies for ALDH expression and DFS/PFS. ALDH, Aldehyde dehydrogenase; DFS, disease-free survival; PFS, progression-free survival

The Begg’s funnel plot and Egger’s test presented no publication bias in IRS of patients with ovarian cancer across the included studies (Begg’s test, *P* = 0.655; Egger’s test, *P* = 0.189) (Fig. 8).

### Sensitivity analysis of all the studies assessing DFS/PFS

**Fig. 9:**
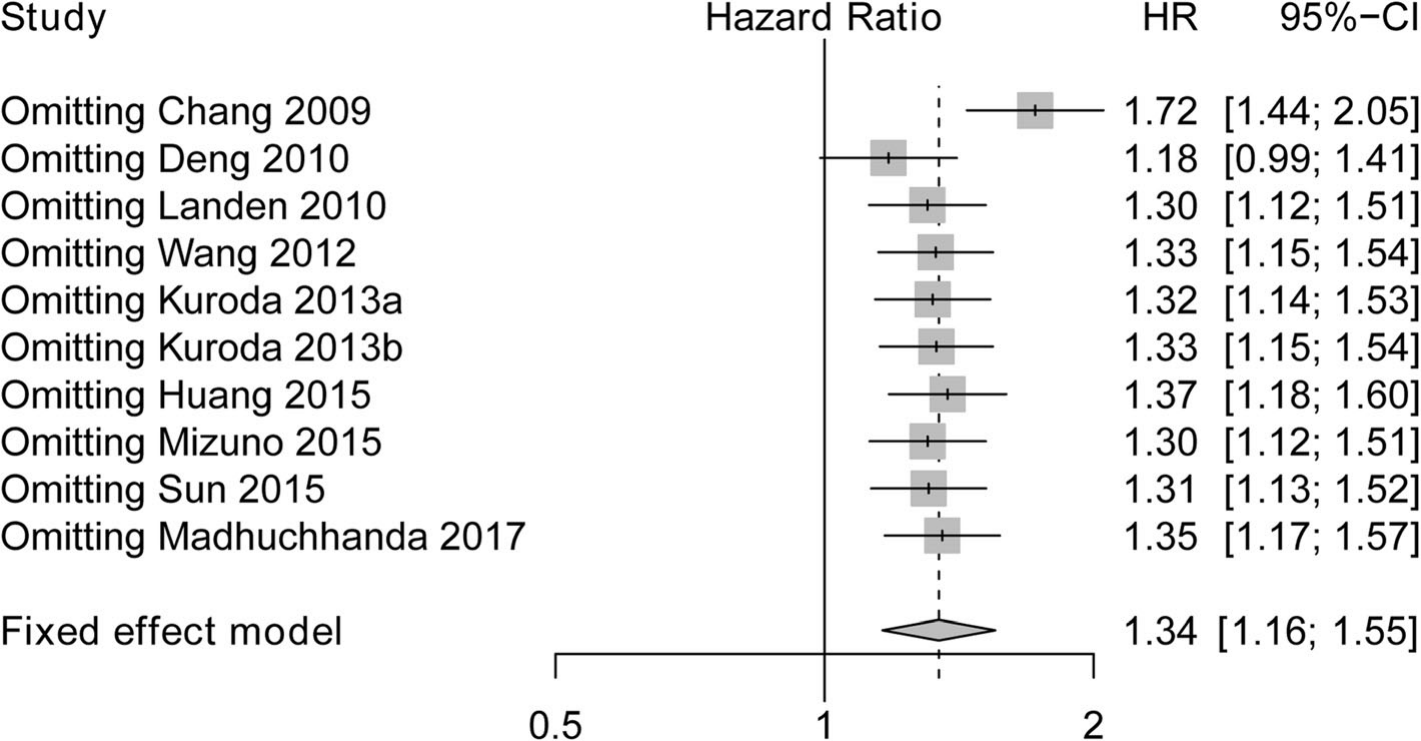
Sensitivity analysis for evaluating the correlation between ALDH expression and DFS/PFS. ALDH, Aldehyde dehydrogenase; HR, Hazard ratio; CI, confidence interval; DFS, disease-free survival; PFS, progression-free survival

Sensitivity analysis revealed that pooled HR of DFS/PFS was not impacted by the exclusion of any single study, although omitting Chang 2009 had a numerically great influence on IRS compared to omitting other studies (Fig. 9).

## Discussion

Malignant tumor has been identified to be consisted of a heterogeneous population of cells, among which, CSCs are a small group of cells presenting with high tumor-initiating potential, which devote into high tumor recurrence and worse distant metastasis in patients with various carcinomas, implying that the elimination of CSCs is essential for improvement of prognosis in these cancer patients [11]. In order to distinguish CSCs from a larger number of cancer cells, there are different methods including utilization of markers in cells surface (such as CD44, CD 90 or CD133), side population (SP) assay as well as ALDEFLUOR assay related to ALDH1 enzyme activity [23–26].

As a common marker of CSCs, ALDH possesses the responsibility to catalyze the oxidation of aldehyde, subsequently contributing to cellular homeostasis, which has been identified to be related to the function as stem cells such as self-renewal capability and stress-resistant properties [7, 25]. Although upregulation of most CSCs markers plays an important role in worse prognosis in cancer patients, several contradictions still exist on the putative CSC-marker ALDH expression in published studies. In order to clarify this problem, only one previous meta-analysis has examined the influence of ALDH on the survival of ovarian cancer patients and discloses that elevated ALDH expression is an independent risk factor for prognosis in ovarian cancer patients [14]. However, that previous meta-analysis just includes 7 studies (6 articles) with a total of 1258 ovarian cancer patients and is published in 2013, whose sample size is relatively small and the published time is relatively early. Therefore, in this comprehensive meta-analysis, we analysed 14 studies (13 articles) with 2210 ovarian cancer patients, among these, 6 studies revealed that high expression of ALDH was associated with poor OS, 4 studies concluded that ALDH expression was positively correlated with DFS/PFS, and Deng et al. showed ALDH was unfavorable factors on both OS and DFS/PFS in ovarian cancer patients [12, 27–34]. However, there were 4 studies that support no association of ALDH with the survival of ovarian cancer [7, 35–37].

In addition, subgroup analysis by different cut-off class also presented with several interesting discovers, which showed correlation of high ALDH expression with poor OS and DFS/PFS when studies set the cut off class as low expression, but not high expression in ovarian cancer patients, which implied that ALDH expression existed in a small subpopulation of cancer cells, and we guessed when the cut-off class is defined as high expression of ALDH, most of ovarian cancer patients with intermediate ALDH expression are excluded and assigned to low expression group.

In this meta-analysis, We performed funnel plot and sensitivity analysis to assess publication bias and the stability of the crude results, and we discovered no evidence of publication bias in OS as well as DFS/PFS of patients with ovarian cancer, and pooled HR of OS as well as DFS/PFS was not impacted by the exclusion of any single study, although omitting Chang 2009 had a numerically great influence on IRS compared to omitting other studies, thus, the conclusion was stable. However, further menta-analysis including new relevant studies is greatly needed for validation.

The underlying mechanism of the correlation between evaluated ALDH expression and worse prognosis in ovarian cancer is still unclear. However, there are several relevant experiments disclosing that ALDH contributes to the processes of tumor development and progression through the influence on cells proliferation, cells apoptosis, cells migration as well as cells invasion. For instance, an interesting experiment displays that ALDH (hi)CD44(+)CD24(−) and ALDH (hi)CD44(+)CD133(+) cells present with enhanced tumorigenicity and metastasis compared to ALDH(low)CD44(low/−) cells, indicating that high ALDH severs as an important role in the enhanced malignant and metastatic ability of breast cancer cells [38]. Another in vitro study reveals that ALDH (high) CD44(+) cells exhibit a higher proliferative, clonogenic and metastatic capacity compared to ALDH(low) CD44(−) cells, suggesting that ALDH has higher tumorigenicity capacity in prostatic cancer [39]. Therefore, ALDH might act as an oncogene in several carcinomas.

Although some interesting results were found in this meta-analysis, some limitations still existed. At first, there were 14 studies included in this comprehensive meta-analysis, and the numbers of studies were relatively small. Meanwhile, all ovarian cancer patients from this meta-analysis were just from five countries (including China, USA, Germany, Japan as well as Norway). In addition, significant heterogeneity still existed partly due to different characteristics of subjects, different cut off value of ALDH expression or other factors, which might lead to confounding bias. Furthermore, subgroup analyses of studies with other characteristics such as histological type, study region or follow-up duration were not carried out. Further meta-analysis included larger-scale studies is necessary.

## Conclusions

In conclusion, high expression of ALDH is correlated with worse survival profiles in ovarian cancer patients, indicating that ALDH might act as a potential molecular biomarker for prognosis of ovarian cancer.

## Abbreviations

ALDH: Aldehyde dehydrogenase
CI: Confidence interval
CSCs: Cancer stem cells
DFS: Disease-free survival
HR: Hazard ratio
IHC: Immunohistochemistry
NA: Not available
NCBI: National Center for Biotechnology Information
OS: Overall survival
PFS: Progression-free survival
PRISMA: Preferred Reporting Items for Systematic reviews and Meta-Analyses
SP: Side population
